# Reported symptoms and patterns of language impairment in bilingual speakers with primary progressive aphasia: a retrospective study

**DOI:** 10.1080/02687038.2026.2691169

**Published:** 2026-06-27

**Authors:** Jessica de Leon, Isabel Elaine Allen, Zachary Miller, Nina Dronkers, Maya Henry, Maria Luisa Gorno-Tempini, Stephanie Grasso

**Affiliations:** a Department of Neurology, Memory and Aging Center, University of California, CA, USA;; b Department of Epidemiology and Biostatistics, University of California, San Francisco, CA, USA;; c Department of Psychology, University of California, Berkeley, CA, USA;; d Department of Communication Sciences and Disorders, University of Texas, Austin, TX, USA

**Keywords:** Bilingualism, primary progressive aphasia, frontotemporal dementia, Alzheimer’s disease

## Abstract

**Background:**

The relative scarcity of studies of bilingual speakers with primary progressive aphasia (PPA) leads to knowledge gaps regarding their symptoms and patterns of language impairment. It is unknown if PPA symptoms in bilingual speakers differ from those of monolingual speakers. In addition, it remains unclear if one of a bilingual speaker’s languages is more vulnerable to the onset of symptoms and/or is better preserved. Furthermore, bilingualism factors (e.g. order/age of language acquisition (L1/L2) and language dominance) may explain patterns of impairment both within and across the variants of PPA.

**Aims:**

To characterize the bilingualism factors, speech and language symptoms, and patterns of language impairment in a retrospective cohort of bilingual speakers with PPA.

**Methods:**

In a large cohort (*N* = 69) of bilingual speakers, we performed a chart review to extract information regarding self/caregiver-reported PPA symptoms, first language impacted by PPA at symptom onset, less preserved language at time of evaluation, and bilingual history factors (age/order of acquisition (L1/L2) and language dominance). We explored how emergence and presentation of symptoms associated with bilingualism factors.

**Results:**

The presenting symptoms were mostly consistent with current PPA diagnostic criteria, although symptoms unique to bilingual speakers were reported. The language reported to first be impacted by PPA, as well as the less preserved language at the time of evaluation, was generally reported to be the less dominant language, regardless of PPA variant. These measures did not associate as closely with age/order of acquisition (L1 versus L2).

**Conclusion:**

Bilingual speakers with PPA may report additional symptoms that reflect their ability to speak more than one language. The less dominant language was most susceptible to the initial impact of PPA and also tended to the less preserved language at the time of evaluation. Future studies of bilingual speakers with neurodegenerative diseases should systematically consider bilingualism factors, which are likely to contribute to clinical presentation and disease progression.

## Introduction

Although the majority of the world’s population speaks two or more languages (Grosjean, 2010; Marian & Shook, 2012), studies on neurodegenerative disease in bilingual speakers are relatively scarce (Franzen et al., 2023). Studies of bilingual individuals presenting with neurodegenerative disease are important, as they may yield crucial knowledge regarding the diagnosis, symptom progression, and treatment of these disorders that is specific to bilingual speakers. In addition, these studies can help elucidate the neurobiology of language organization in the bilingual brain. That is, because there are relatively stable patterns of atrophic changes in neurodegenerative syndromes such as primary progressive aphasia (PPA), one can infer the degree to which the languages of a bilingual speaker share the same underlying neural substrates on the basis of observed patterns of language decline and impairment.

Several factors likely play a role in the neurocognitive organization of the bilingual brain (DeLuca et al., 2019; Pliatsikas, 2020), and we focus on two of these factors in our current restrospective study. Since there is variability across studies in how these bilingualism experiences are measured, we describe our use of them here:

1. Age/order of acquisition (hereafter referred to as “age of acquisition” or AoA for simplicity) allows us to examine patterns on the basis of earlier vs. later acquired languages. Studies including age of acquisition typically refer to L1 as the first language acquired by an individual, whereas L2 is the second language acquired over the individual’s lifetime.
2. Language dominance is a more global measure that may include several components of the bilingual experience and is sometimes described as the most proficient language or stronger of the two languages (Treffers-Daller, 2019). In some measures of language dominance aspects such as language/cultural identity, confidence and use may be incorporated (Snape & Kupisch, 2016).

While L2 does not change because it is determined by the age at which the person acquires a language, an individual’s language dominance can change over time.

Most studies to-date that have investigated language decline in bilingual speakers with neurodegenerative syndromes have focused on Alzheimer’s dementia (Calabria et al., 2017; Gómez-Ruiz et al., 2012; Manchon et al., 2015; Mario et al., 1999; Meguro et al., 2003; Smirnov et al., 2019; Stilwell et al., 2016). Fewer studies have investigated patterns of language impairment in language-prominent dementia syndromes such as primary progressive aphasia (PPA) (Costa et al., 2019; Ellajosyula et al., 2020; Malcolm et al., 2019). Previous PPA studies suggest that the less dominant or least used language prior to disease onset may be the first to be impacted by symptoms, regardless of whether the language was L1 or L2, but there is heterogeneity across studies (Costa et al., 2019; Filley et al., 2006). Other studies that have examined order of language acquisition generally find that the second language learned (L2) is the most affected, or they observe “parallel”; (e.g., equivalent) decline of both languages (Costa et al., 2012, 2019; Ellajosyula et al., 2020; Larner, 2012; Machado et al., 2010; Malcolm et al., 2019; Mendez et al., 2004). Nevertheless, it is not well understood how bilingualism factors associate with patterns of language decline in PPA variants (Costa et al., 2019; Filley et al., 2006).

In the present study, we focus on PPA (Gorno-Tempini et al., 2011; Mesulam, 1982), which has been characterized into three main clinical variants: 1) non-fluent/agrammatic variant primary progressive aphasia (nfvPPA), which is characterized by agrammatism and motor speech deficits, left inferior frontal atrophy, and typically frontotemporal lobar degeneration (FTLD)- 4 R tau pathology (Grossman, 2012; Josephs, 2006; Ogar et al., 2007); 2) semantic variant primary progressive aphasia (svPPA), which presents with loss of object knowledge, anomia, and surface dyslexia/dysgraphia; anterior temporal atrophy on imaging; and FTLD-TDP-43-C pathology in most cases (Hodges et al., 1992); and 3) logopenic variant primary progressive aphasia (lvPPA), which is characterized by phonological deficits, impaired repetition, and anomia with left temporo-parietal atrophy on imaging and Alzheimer’s disease pathology in most cases (Gorno-Tempini et al., 2004; Santos-Santos et al., 2018). Because this group of disorders varies in their speech and language symptoms and underlying affected neural networks, studies of bilingualism across the PPA variants could give insights into how different domains of speech and language may either overlap or diverge in a bilingual speaker’s brain, depending on the patterns of language loss in patients with PPA. The characterization of bilingual PPA may also yield findings that can help refine current frameworks describing the neural representation of languages in bilingual speakers and may guide clinical care and treatment of these individuals.

The purpose of this current study is to explore how two bilingualism history factors (i.e., age/order of acquisition, language dominance) are associated with subjective report of PPA symptoms and patterns of impairment. To achieve this, we 1) characterize bilingualism factors in a retrospective cohort of bilingual speakers with PPA, 2) describe first PPA symptoms as reported by the patient and/or their caregiver, and 3) describe how bilingualism factors (L1 vs. L2, and dominant vs. nondominant) relate to their PPA presentation. We report the *first language impacted* (defined as the language in which symptoms were first noticed by the participant and/or caregiver) and the *less preserved language* (defined as the language that is noted to have experienced most decline by the participant and/or caregiver at the time of diagnosis). We address the following hypotheses:

1. First self-reported symptoms: Bilingual speakers might report additional PPA symptoms that could be unique to those who speak more than one language and may not be fully captured in the current PPA diagnostic criteria.
2. Language first impacted: Language dominance plays a stronger role than age/order of language acquisition in determining the *first language impacted* (Costa et al., 2019; Ellajosyula et al., 2020; Filley et al., 2006; Larner, 2012; Machado et al., 2010; Malcolm et al., 2019; Mendez et al., 2004).
3. Language less preserved at time of diagnosis: In alignment with hypothesis 2, Language dominance plays a stronger role than age/order of language acquisition in *language preservation* over time, in accordance with previous studies (Costa et al., 2019; Ellajosyula et al., 2020; Filley et al., 2006; Larner, 2012; Machado et al., 2010; Malcolm et al., 2019; Mendez et al., 2004).

As this study is based on retrospective data, we were restricted to examining the available information on bilingualism from patient charts. However, since bilingualism factors are not often reported in PPA studies, even despite known effects of bilingualism on multiple aspects of language functioning and cognitive testing performance (Calabria et al., 2018; Celik et al., 2022; Costa & Sebastián-Gallés, 2014; Grundy et al., 2017; Rivera Mindt et al., 2008; Tamminen et al., 2013), this study sought to provide further rationale for the inclusion of measures characterizing bilingualism in the study of PPA.

## Methods

### Participants

Participants were recruited through a parent longitudinal research program at the UCSF Memory and Aging Center (MAC). As part of the research protocol, all participant visits included a detailed clinical history obtained from the participant and caregiver, a neurological examination, and a caregiver interview to assess functional status. Participants also received comprehensive neuropsychological testing (Kramer et al., 2003) (Supplemental Table 3) and a speech and language battery (Supplemental Table 4). A multidisciplinary team met and assigned a diagnosis to each participant using the same established PPA diagnostic criteria (Gorno-Tempini et al., 2011). At the end of each visit, a written research visit summary was generated, containing comprehensive data from these study components (Gorno-Tempini et al., 2011; Rascovsky et al., 2011). For the purposes of this cross-sectional study, we included information only from the first visit. Each participant and/or their decision-making surrogate provided written consent for the parent study, which was approved by the UCSF institutional review board for human research.

### Determination of monolingual or bilingual status

A comprehensive chart review was performed to identify bilingual speakers for inclusion in this study, using procedures outlined previously (de Leon et al., 2020). Research visit summaries from the UCSF MAC database were queried for terms related to bilingualism (see Supplementary Table 1 for a full list of search terms). Two trained reviewers (JD and SG) then extracted this information from the visit summaries. Patients were classified as bilingual speakers if the chart indicated that they could communicate in two or more languages in everyday interaction with other speakers of these same languages (Alladi et al., 2017; de Leon et al., 2023; Emmorey & McCullough, 2009; Grosjean, 2010; Mohanty, 1994); see Supplemental Materials for additional details.

Percent agreement rates were used as a measure of inter-rater reliability. To establish inter-rater reliability, each of the two scorers independently applied the same assessment criteria. First, each rater evaluated the available information for each participant and determined if they met criteria for bilingual speakers, as outlined in the Supplementary Materials. If so, the participant was classified as bilingual. Second, if the available information met the exclusion criteria listed in the Supplementary Materials, or there was no other information to support a diagnosis of bilingualism based on the criteria, the participant was classified as a monolingual speaker. Therefore, at the end of this process, each individual had one of two classifications: bilingual or monolingual. This classification was then compared between the two reviewers. If there was a discrepancy between the classification, this was recorded as a mismatch when determining the percent agreement rates. Overall, there was 90% inter-rater reliability. In cases of disagreement or uncertainty, the raters conferred to reach consensus.

The chart review yielded 69 bilingual participants total who met diagnostic criteria for one of the three main PPA variants (Gorno-Tempini et al., 2011). The charts of this final cohort underwent a more thorough data extraction process to determine the first acquired language (L1), second acquired language (L2) and any additional languages spoken by the individual, age of acquisition of L2, context for L2 learning (e.g., formal education, immigration, home or work environment), dominant oral language, immigration history, and occupation (ISCO-08) (Office, IL [IL], 2012). This data was obtained from interviews with the patient and caregiver during the study visit. When available, we extracted information collected from the participant and/or caregiver regarding reported speech and language symptoms, the subjective report of the language that was first affected (e.g., L1 versus L2, or dominant vs. nondominant language), and the language perceived to be the less preserved at the time of evaluation. This information was derived from responses given when the participant and/or caregiver were asked by the neurologist about observed changes in speech and language and then categorized accordingly. Demographic information, including sex, education, handedness, age at first symptom, age at UCSF MAC evaluation, and clinical diagnoses were available through an internal MAC database.

### Statistical analyses

For general characterization of the study participants, we provide descriptive statistics summarizing demographic variables for the full cohort and by PPA variant. To address our first question: *What are the first self-reported speech-language symptoms in bilingual speakers with PPA?*, we summarize the frequency of first observed PPA symptoms. *To address our second question: Which language is reported to be the first impacted in bilingual speakers with PPA?*, we summarize bilingual history factors and the frequency of observed patterns of language impairment (i.e., self/caregiver-reports of first language impacted). *To address our third question: 3) Which language is reported to be the less preserved language in bilingual speakers with PPA?*, we summarize the frequency of reports indicating which language was less preserved at the time of evaluation. In the section that follows, we report results organized by these three research questions subsequent to providing demographic characteristics of our participants.

## Results

### Demographic data

The study included a total of 69 participants. The cohort was 55% female. The mean age of education was 16.7 years (*SD* = 2.7). The mean age at symptom onset was 62.1 years, with a mean age at evaluation of 67.1 years (Table 1). Of the 69 participants, 22 were diagnosed with nfvPPA, 31 with svPPA, and 16 with lvPPA based on clinical symptoms (Table 2). The majority of cases were also imaging supported (17/22 (77%) of nfvPPA, 29/31 (94%) of svPPA, and 9/16 (56%) of lvPPA). The bilingual speakers spoke a variety of languages (see Supplementary Table 2). English was the L1 (first language acquired) for 29 participants, L2 for 32 participants, and L3 for 8 participants.

### General characterization of baseline bilingualism profiles

The bilingualism profiles including bilingualism-related factors of the participants are reported for the entire PPA cohort and by PPA variant in Table 3. Across the PPA variants, 41% were considered to be “early” bilingual speakers. For the purposes of this study, this was operationally defined as an individual who learned L2 prior to age 7 (Byers-Heinlein & Lew-Williams, 2013; Johnson & Newport, 1991; Kuzmina et al., 2019). The participants spoke an average of 2.7 languages, and 51% were multilingual (spoke three or more languages). The average L2 age of acquisition was 12.1 years (range 1–38 years). There were several contexts for L2 learning, including formal education (59%), immigration (24%), home environment (12%), and work (2%). Approximately half of the cohort (49%) indicated that they had immigrated to another country, with an average age at immigration of 19.4 years.

Most of the cohort (69%) consisted of lifelong bilingual speakers who were continuing to regularly use two or more languages at the time of evaluation. Participants also reported varying patterns of language use, with approximately half of the cohort reporting use of L1 more than L2 (48%), and fewer participants reporting use of L2 more than L1 (34%), or equal use of both languages (17%). The most dominant language was L1 in the majority of cases (74%) and L2 in the minority of cases (26%).

### Patterns of subjective report of language impairment in the context of bilingualism factors

Question 1: *What are the first self-reported speech-language symptoms in bilinguals with PPA?*: The first reported symptoms consistent with PPA are reported in Table 4. For nfvPPA participants, the most commonly-reported first symptoms were effortful speech/difficulty with pronunciation (67%), word-finding difficulties (33%), word subsitutions (10%), and phonemic paraphasias (10%) (e.g., “He started making phonemic errors such as saying ‘spolish’ when he means ‘polish’”). In svPPA, the majority of participants reported word-finding and naming difficulties (72%) and far fewer reported difficulty with comprehension (31%). In lvPPA, the majority of participants reported word finding difficulties (75%) and far fewer reported difficulty with comprehension (25%). In addition, in the minority of cases, the initial symptoms observed are considered to be unique to bilingual speakers, including difficulties with code switching (e.g., “She will mix Chinese and English even within one word”) and difficulty translating (e.g., “about a year and a half ago on a trip to Italy [participant’s L1 is Italian] she noted that he had trouble translating”).

**Figure 1.**
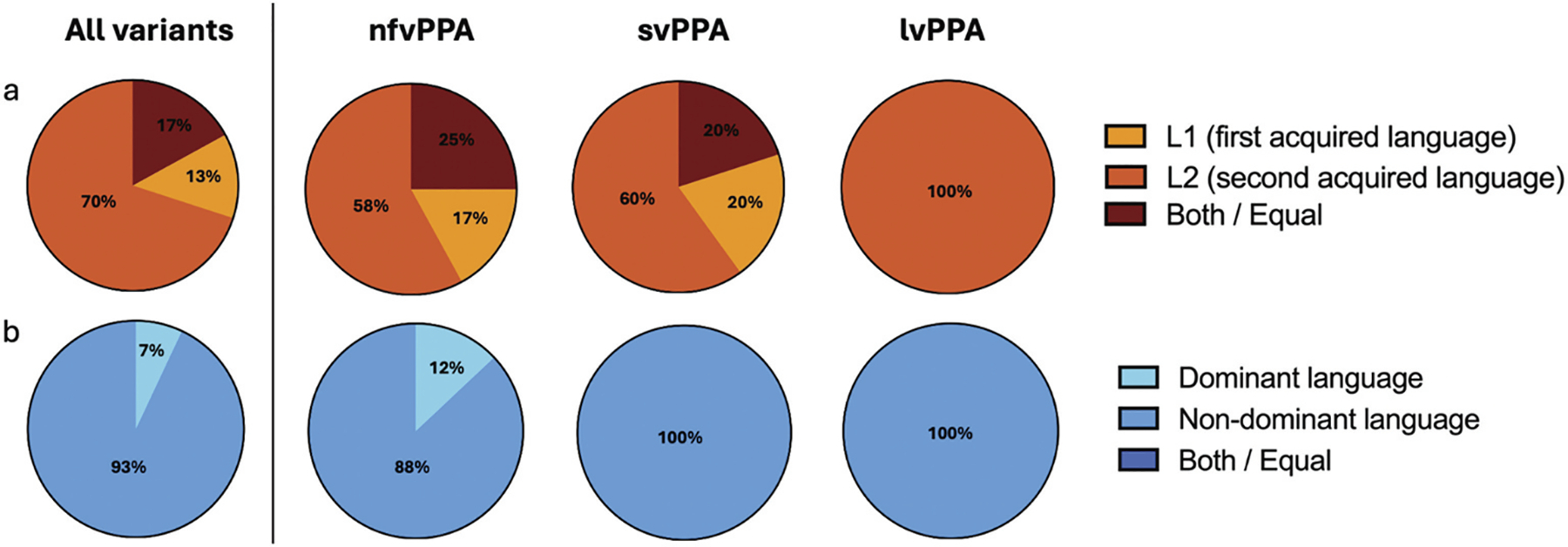
First language impacted: by L1/L2 and by language dominance.

Question 2: *Which language is reported to be the first impacted in bilingual speakers with PPA?*: When assessing first language impacted by PPA based on L1 (first acquired language) versus L2 status, 70% of participants across the PPA variants reported symptoms first in L2, 13% reported first symptoms in L1, and 17% reported that both languages were affected. Within the nfvPPA and svPPA cohorts, participants differed in whether L1, L2, or both were the first language impacted, while in the lvPPA cohort, L2 was reported to be the first language impacted in all cases (Figure 1(a)). Interestingly, when examining language dominance, all participants except one nfvPPA participant reported that their less dominant language was the first language impacted by PPA, regardless of whether this language represented their L1 or L2 (Figure 1(b)).

**Figure 2.**
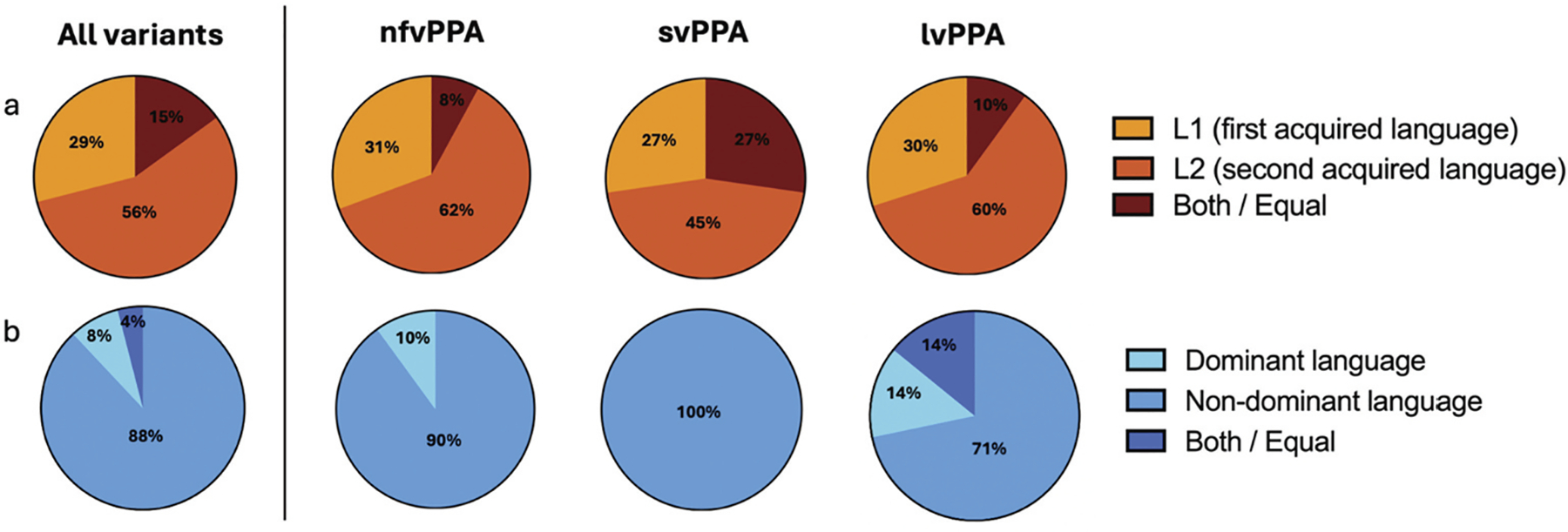
Less preserved language at time of evaluation: by L1/L2 and by language dominance.

Question 3: *Which language is reported to be the less preserved language in bilinguals with PPA?*: When considering patterns based on age/order of language acquisition, in slightly more than half of cases (56%), L2 was the less preserved language at the time of diagnosis. When looking within variants, a higher percentage of svPPA participants reported that both languages were affected equally relative to the other PPA variants (27% in svPPA, compared to 8% in nfvPPA and 10% in lvPPA) (Figure 2(a)). When considering language dominance, the majority of participants (88%) reported relatively less preservation of their non-dominant language compared to their dominant language, and this was similar within each variant (Figure 2(b)).

## Discussion

To our knowledge, this is the largest descriptive study to-date of symptoms and patterns of language impairment in bilingual individuals with PPA. The goals of our study were to characterize the bilingualism factors and first PPA symptoms of the cohort and to describe how bilingualism factors associate with symptom emergence and presentation. This study adds to current knowledge of PPA in bilingual speakers, who have been historically underrepresented in the literature, and helps advance our understanding of PPA in culturally and linguistically diverse populations.

### Clinical symptoms at presentation

Across PPA variants, the presenting symptoms tended to be consistent with the symptoms outlined in the current PPA diagnostic criteria. It is notable, however, that in some cases, the first reported symptom was one that would be unique in a bilingual speaker, including code switching in one participant with nfvPPA and difficulty translating for one participant with lvPPA. Together, these observations suggest the need to be attuned to speech and language symptoms in bilingual speakers that may not be fully captured in the current diagnostic criteria, as they are largely centered on impairments characterized in monolingual English speakers.

Additional research is also needed to identify the symptoms of PPA that may manifest differently in an individual’s L1 or L2. This may depend on the specific language typology and may manifest differently in speakers of different language combinations (Canu et al., 2020; Tee et al., 2021, 2022). Since the participants in our study were all evaluated in English, and available assessment measures for PPA are limited in many of their languages, it is unclear if their PPA symptoms and diagnoses would have varied in their L1 or L2. Even further, patterns of language deficits may additionally vary in trilingual and multilingual speakers, and these patterns have not yet been well-characterized. While there have been important contributions on this topic from case reports (Calabria et al., 2021; Kambanaros & Grohmann, 2012; Kelly & Shehab, 2025; Liu et al., 2012; Mendez et al., 2004), it remains relatively unknown whether a third language (L3) could provide additional support against language decline; whether L2 impairment is more similar to L1 or L3; and how patterns of decline are affected by age at acquisition, proficiency and/or patterns of language use of L3. To improve diagnosis and further explore this possibility, future projects should move towards evaluation in both (or all) of a bilingual speakers’ languages (Russell-Meill et al., 2025; Sung et al., 2024). This would necessitate the creation and adaptation of PPA assessment measures for a wider range of languages, also taking into account language typology and features of PPA that may vary depending on the language (Arslan & Peñaloza, 2026; Franzen et al., 2023; García et al., 2023).

Another area of consideration is the effect of bilingualism on cognitive reserve. Previous studies suggest that speaking two or more languages may serve as a protective factor against neurodegenerative diseases and may manifest as a later age of symptom onset (Alladi et al., 2013; Bialystok et al., 2007, 2014; Mendez et al., 2019), improved functioning on executive control tasks (Bialystok, 2011; Celik et al., 2020), or slower cognitive and functional decline (Costumero et al., 2020; Tablante et al., 2025), although effects of bilingualism in PPA may depend on the variant (Alladi et al., 2017; de Leon et al., 0000, 2023). Future studies should consider whether bilingualism may impact age or severity at which clinical symptoms are perceived, including symptoms that may be unique to bilingual speakers, or whether bilingualism may mask detection of symptoms in other domains, including executive functioning.

### First language impacted in bilingual speakers with PPA

In general, we found that individuals with PPA reported changes in both of their languages, consistent with previous studies and models that point to at least partially shared processing between languages (Calabria et al., 2017; Druks & Weekes, 2013; Illes et al., 1999; Sulpizio et al., 2020). The first language impacted across all PPA variants was usually the non-dominant language (93% of cases overall), regardless of whether this was L1 (the first acquired language) or L2 (the second acquired language). On the other hand, when examining the relationship between age/order of language acquisition (L1 vs L2) and first language impacted, there was slightly more variability, such that in the majority of cases (70%), L2 (the second acquired language) was the first language impacted, although some participants did notice symptoms first in L1 or in *both* languages simultaneously. This pattern mirrors that of previous studies in neurodegenerative disease, in that language changes may depend more on the dominant (most proficient) language rather than the order of acquisition (L1 vs L2) (Ellajosyula et al., 2020; Smirnov et al., 2019). However, it should be noted that the dataset for our study allowed for mainly descriptive analyses, limiting the interpretation of these findings.

When examining patterns within PPA variants, some notable patterns emerged. While there was relative consistency in the non-dominant language being affected first, there was more variation in the first language impacted when considering order of acquisition (L1 vs L2). Within the lvPPA cohort, all participants first noted symptoms in L2 (second acquired language), whereas this was more heterogeneous in nfvPPA and svPPA. It should be emphasized that the descriptive nature of our study cannot directly address neuroanatomical patterns. With this in mind, this finding could be considered in light of past studies that have shown that the phonological networks of a bilingual speaker’s two languages have lesser degrees of overlapping structure and connectivity compared to other language processes such as semantics and morphosyntax (Richardson & Price, 2009; Sebastián-Gallés et al., 2012). As such, one might speculate that our finding that L2 was affected first in all our lvPPA cases could potentially reflect a lesser degree of neuroanatomical overlap that exists between L1 and L2 phonological networks, leaving L2 more vulnerable to decline at earlier stages of disease. However, given that our broader cohort showed a consistent pattern of the nondominant language being affected first, it remains unclear whether the uniform L2 involvement in lvPPA reflects phonological network properties or simply the heightened vulnerability of a less dominant language, regardless of whether it is L1 or L2. To more adequately evaluate this, future studies should be conducted in larger datasets, with more detailed information regarding language acquisition and language dominance, among other bilingualism factors.

### Less preserved language in bilingual speakers with PPA

Similar to the pattern observed for first language impacted, the less preserved language tended to reflect the non-dominant language (88% of cases overall across the PPA variants), with more variation in whether this represented the order of language acquisition (L1 vs L2), such that the less preserved language could represent L1 (29%), or L2 (56%), or both equally (15%). Our finding is therefore in line with previous studies showing that the pattern of language preservation is more strongly tied to the more dominant language rather than the first learned language (Ellajosyula et al., 2020; Smirnov et al., 2019).

When comparing bilingual speakers within the three PPA variants, there were similar patterns in the proportion of speakers with reported preservation of either L1 or L2, but interestingly there was a higher percentage of speakers in the svPPA group who reported that both languages were affected equally (27% in svPPA vs. 8% in nfvPPA and 10% in lvPPA). One potential explanation for the higher percentage of equal decline of both languages in svPPA is that there is a greater degree of overlap in networks supporting semantic processing. This would be in line with the main models of organization of semantic knowledge (Dijkstra & van Heuven, 2002; Kroll & Stewart, 1994; Van Hell & De Groot, 1998) as well as past studies (Illes et al., 1999; Klein et al., 1995) that posit a high degree of overlap of semantic systems in bilingual speakers. Several models describe a shared conceptual store across languages; therefore, should semantic knowledge begin to erode, such as in the case of svPPA, relatively equivalent impairment would be anticipated in this domain (Francis, 1999; Kroll et al., 2010). Overall, while our study can provide descriptive findings in a cohort of patients with PPA variants, larger studies will allow for more rigorous testing of these potential patterns.

### Strengths and limitations

Strengths of our study include the relatively large sample size of bilingual PPA patients. Since this study was performed at a tertiary care center, there was also the opportunity for careful phenotyping of symptoms and expert diagnosis by a team of behavioral neurologists, neuropsychologists, speech language pathologists, and nursing staff.

There are several important limitations and considerations. Despite the relatively large cohort of the current study, our sample sizes within PPA subgroups were relatively small and unbalanced. As such, larger scale collaborative endeavors will be necessary to more thoroughly investigate patterns of PPA symptoms within the variants of PPA.

In addition, in the current study we were able to extract information regarding global perceptions of language decline (e.g., did individuals report greater decline in one language vs. another). To build on this, future research should prospectively examine patterns of language impairment and decline on the basis of various linguistic domains (e.g., lexical-semantics, morphosyntax, phonology) within each language (including third or fourth languages, if applicable), and within each PPA variant. Larger studies examining impairment profiles and decline in bilingual PPA are warranted to replicate and extend the patterns reported herein.

Future studies should also examine patterns of language decline objectively through neuropsychological testing. In order to accomplish this in an appropriate manner, there is an immense need to develop cognitive batteries in a wider range of languages and to improve the availability of clinicians who speak diverse languages. To emphasize this, to our knowledge, there is a lack of cognitive batteries to assess for PPA in several of the languages spoken by our cohort, which impedes the ability to perform testing in all of a patient’s languages and obtain a full picture of potential strengths and deficits. This also creates challenges in being able to fully assess potential effects of language typology (e.g., differences in morphology, orthography, phonology) that may lead to differences in symptoms across languages or in patterns of decline. Furthermore, in the United States, there is a scarcity of neuropsychologists who are bilingual (Elbulok-Charcape et al., 2014; Rivera Mindt et al., 2010), further hindering the availability of language and culturally-concordant evaluations.

Finally, the retrospective nature of this study contributed to limitations in the available data regarding bilingualism factors and initial clinical symptoms. The study relied on chart review data, and information regarding bilingualism (e.g., language dominance) was not reported in a systematic manner by the clinicians. The dataset for this study spans charts from a period of more than 20 years. For the majority of the research program’s protocol during this time, data related to bilingualism was not collected in a consistent fashion. Historically, there has been a lack of inclusion of this type of data in PPA cohorts and in dementia cohorts more broadly. The patterns rerported herein highlight the need to collect detailed information regarding bilingualism factors (e.g., L2 age of acquisition, manner of L2 acquisition, patterns of language use) in a systematic manner. We fully acknowledge that bilingualism is a nuanced and complex life experience, and these bilingualism factors certainly contribute to clinical presentation.

## Conclusion

In this study, we identified the first-reported symptoms and patterns of L1 vs L2 language impairment in a relatively large cohort of individuals with bilingual PPA. The results of our study indicate that language dominance prior to symptom onset is the best indicator both for which language will be impacted first in PPA and for which language will be less preserved, with the non-dominant (less proficient) language demonstrating greatest vulnerability. While the results should be interpreted cautiously in the context of being a retrospective, clinically-based study, this study contributes to the field’s knowledge of the patterns of symptoms and language decline reported in bilingual individuals with PPA, a much-needed area of investigation given the world’s substantial and growing population of bilingual speakers.

## Supplementary Material

Supp 1

Supplemental data for this article can be accessed online at https://doi.org/10.1080/02687038.2026.2691169

## Figures and Tables

**Table 1.** Demographic information for bilingual speakers (full cohort).

	Bilingual speakers (*N* = 69)
Sex, Female, n (%)	38 (55)
Education, mean (SD), y	16.7 (2.7)
Right-handed, n (%)	60 (87)
Occupation	
Professionals, n (%)	43 (62)
Associate professionals, n (%)	12 (17)
Skilled workers, n (%)	14 (20)
Elementary, n (%)	0 (0)
Immigrant, n (%)	28 (41)
Age at onset, mean (SD), y	62.1 (7.6)
Age at evaluation, mean (SD), y	67.1 (7.7)

**Table 2.** Demographic information for bilingual speakers by clinical syndrome.

	nfvPPA (*N* = 22)	svPPA (*N* = 31)	lvPPA (*N* = 16)
Sex, Female, n (%)	15 (68)	18 (58)	5 (31)
Education, mean (SD), y	16.9 (2.2)	16.7 (2.8)	16.3 (3.3)
Right-handed, n (%)	20 (91)	27 (87)	13 (81)
Occupation			
Professionals, n (%)	15 (68)	18 (58)	10 (63)
Associate professionals, n (%)	3 (14)	6 (19)	3 (19)
Skilled workers, n (%)	4 (18)	7 (23)	3 (19)
Elementary, n (%)	0 (0)	0 (0)	0 (0)
Age at onset, mean (SD), y	**63.6 (7.1)**	**60.3 (7.8)**	**63.6 (7.4)**
Age at evaluation, mean (SD), y	**67.6 (7.8)**	**66.1 (7.2)**	**68.2 (8.6)**

**Table 3.** Features of bilingual patients by variant.

	nfvPPA (N = 22)	svPPA (N = 31)	lvPPA (N = 16)	All variants (N = 69)
Early bilingual speakers	5/16 (31%)	10/28 (36%)	8/12 (67%)	23/56 (41%)
Total number of languages	2.7 languages 9/22 (41%) multilingual	2.6 languages 17/31 (55%) multilingual	2.9 languages 9/16 (56%) multilingual	2.7 languages 35/69 (51%) multilingual
L2 age of acquisition	13.5 years	11.9 years	10.1 years	12.1 years
Context for L2 learning	12/15 (80%) formal education 2/15 (13%) immigration 1/15 (7%) home	13/23 (57%) formal education 7/23 (30%) immigration 1/23 (4%) home 1/23 (4%) work	4/11 (36%) formal education 3/11 (27%) immigration 4/11 (36%) home	29/49 (59%) formal education 12/49 (24%) immigration 6/49 (12%) home 1/49 (2%) work
Immigrant status	9/18 (50%)	17/28 (61%)	4/15 (27%)	61 (49%)
Age at immigration	19.6 years	19.3 years	19.0 years	19.4 years
Most used language at time of evaluation	L1 > L2 5/13 (38%) L2 > L1 5/13 (38%) L1 = L2 3/13 (23%)	L1 > L2 6/11 (55%) L2 > L1 4/11 (36%) L1 = L2 1/11 (9%)	L1 > L2 3/5 (60%) L2 > L1 1/5 (20%) L1 = L2 1/5 (20%)	L1 > L2 14/29 (48%) L2 > L1 10/29 (34%) L1 = L2 5/29 (17%)
Dominant language at time of evaluation	L1 10/13 (77%) L2 3/13 (23%)	L1 8/12 (67%) L2 4/12 (33%)	L1 7/9 (78%) L2 2/9 (22%)	L1 25/34 (74%) L2 9/34 (26%)

**Table 4.** First PPA symptoms by PPA variant.

nfvPPA	svPPA	lvPPA
Effortful speech and difficulty with pronunciation 14/21 (67%) Difficulty with grammar 1/21 (5%) Word-finding difficulties 7/21 (33%) Phonemic paraphasias 2/21 (10%) Code switching 1/21 (5%) Word substitutions 2/21 (10%)	Word-finding difficulties 21/29 (72%) Difficulty with comprehension 9/29 (31%) Word substitution/generalization 3/29 (10%) Change in speech production 3/29 (10%)	Word-finding difficulties 12/16 (75%) Difficulty with comprehension 4/16 (25%) Difficulty translating 1/16 (6%) Mispronouncing words 1/16 (6%)

## Data Availability

The data that support the findings of this study are not publicly available due to the conditions of our ethics approval and other patient confidentiality requirements. Access will be granted on request from the corresponding author [JD] through a formal data sharing agreement in accordance with existing institutional procedures.
