# Supplementary material for: Reported symptoms and patterns of language impairment in bilingual speakers with primary progressive aphasia: a retrospective study
Source: Aphasiology. Author manuscript; Available in PMC 2026 Jul 8. (PMC13341156; doi:10.1080/02687038.2026.2691169)
Supplement: Supp 1 [file NIHMS2191953-supplement-Supp_1.docx]

## Supplementary Table 1: List of search terms

Top 20 places of birth for the foreign-born population in the United States

Mexico China India Philippines Vietnam

El Salvador Cuba

South Korea Dominican Republic Guatemala

Canada Jamaica

United Kingdom Indonesia

Colombia Haiti Germany Honduras Peru Poland

Top 100 languages by estimated number of native speakers

Mandarin Spanish English Hindi Arabic Portuguese Bengali Russian Japanese Punjabi German Javanese

Wu (Shanghainese) Malay (Malaysian, Indonesian)

Telugu Vietnamese Korean French Marathi Tamil

Urdu Turkish Italian

Yue (Cantonese) Thai

Gujarati Jin

Southern Min (Hokkien, Teochew)

Persian (Farsi) Polish

Pashto Kannada

Xiang (Hunanese) Malayalam Sundanese Hausa

Odia Burmese Hakka Ukrainian Bhojpuri Tagalog Yoruba Maithili Uzbek Sindhi Amharic Fula Romanian Oromo Igbo Azerbaijani Awadhi

Gan Chinese Cebuano (Visayan) Dutch

Kurdish

Serbo-Croatian Malagasy Saraiki

Nepali Sinhalese Chittagonian Zhuang Khmer Turkmen

Assamese Madurese Somali Marwari Magahi Haryanvi Hungarian Chhattisgarhi Greek Chewa Deccan

Akan Kazakh

Northern Min Sylheti

Zulu Czech

Kinyarwanda Dhundhari Haitian Creole

Eastern Min (Fuzhounese) Ilocano

Quechua Kirundi Swedish Hmong Shona Uyghur

Hiligaynon (Ilonggo, Visayan) Mossi

Xhosa Belarusian Balochi Konkani

Other:

-anish Accent Interpre-Biling-Transla-Immig-Emmig-Second lang-Native Learned Eng-Multiling-Two lang-

Multiple lang-Moved to

# Supplementary Table 2. L1 and L2 for bilingual speakers by variant

| **lvPPA** |  | | | | |
| --- | --- | --- | --- | --- | --- |
| **L1** | **L2** | **Other Languages** | **N** |  |  |
| Cantonese | English | Swedish | 1 |  |  |
| English | French |  | 2 |  |  |
| English | German |  | 1 |  |  |
| English | Greek |  | 1 |  |  |
| English | Hebrew | Spanish | 1 |  |  |
| English | Spanish | French, Italian, Portuguese | 1 |  |  |
| Hebrew | English | French | 1 |  |  |
| Italian | English |  | 2 |  |  |
| Italian | Spanish | English | 1 |  |  |
| Kapampangan | Tagalog | English | 1 |  |  |
| Malayalam | Hindi | English | 1 |  |  |
| Marathi | Hindi | English | 1 |  |  |
| Portuguese | English |  | 1 |  |  |
| Swedish | German | English, Russian, Spanish | 1 |  |  |
| **nfvPPA** | | | |  |  |
| **L1** | **L2** | **Other Languages** | **N** |  |  |
| Cantonese | Taiwanese |  | 1 |  |  |
| Czech | English |  | 1 |  |  |
| English | ASL |  | 1 |  |  |
| English | French |  | 2 |  |  |
| English | French | Spanish | 1 |  |  |
| English | German | Italian | 1 |  |  |
| English | German | Spanish | 1 |  |  |
| English | German | French, Turkish, Russian, Spanish | 1 |  |  |
| English | Spanish |  | 4 |  |  |
| English | Spanish | Yiddish | 1 |  |  |
| Farsi | English |  | 1 |  |  |
| German | English | French | 1 |  |  |
| German | English | French, Latin, Greek | 1 |  |  |
| Mandarin | English |  | 1 |  |  |
| Spanish | English |  | 4 |  |  |
| **svPPA** | | | |  |  |
| **L1** | **L2** | **Other Languages** | **N** |  |  |
| Danish | English | Spanish, French, Latin | 1 |  |  |
| Dutch | English |  | 1 |  |  |
| English | Farsi | Spanish | 1 |  |  |
| English | French | Spanish | 2 |  |  |
| English | German | French | 1 |  |  |

| English | Spanish |  | 4 |
| --- | --- | --- | --- |
| English | Spanish | German | 1 |
| English | Spanish | French, Italian | 1 |
| English | Spanish | Japanese | 1 |
| Farsi | English |  | 1 |
| French | English |  | 1 |
| German | English |  | 2 |
| German | English | Hebrew | 1 |
| German | English | French | 1 |
| Hebrew | Yiddish | English | 1 |
| Latvian | English |  | 1 |
| Mandarin | English | Cantonese | 1 |
| Punjabi | Hindi | English | 1 |
| Swiss German | English | French | 1 |
| Tagalog | English |  | 1 |
| Tagalog | English | Spanish | 1 |
| Tamil | English | German | 1 |
| Russian | English |  | 2 |
| Spanish | English |  | 1 |
| Yiddish | English | German | 1 |

# Supplementary Table 3. Neuropsychological testing battery

| **General** |
| --- |
| Mini Mental State Exam |
| Geriatric Depression Scale (30 item) |
| **Memory** |
| CVLT (9-item) |
| Modified Rey Recall |
| **Language** |
| Sentence Repetition |
| Verbal Agility |
| WRAT-4 Reading |
| Sentence Comprehension |
| PPVT-R Comprehension |
| **Category/Animal Fluency** |
| Boston Naming Test (15 item) |
| **Visuospatial** |
| Modified Rey Copy |
| Calculations |
| VOSP Number Location |
| **Frontal/executive** |
| Modified Trails |
| Design Fluency |
| Alternating m and n |
| Digits Forward |
| Digits Backward |
| Verbal/Phonemic Fluency |
| Abstraction |
| Stroop Color Naming/Interference |

# Supplementary Table 4. Speech and language battery

| **Western Aphasia Battery** |
| --- |
| Spontaneous Speech |
| Picture Description |
| Comprehension |
| Repetition |
| **Motor Speech Evaluation** |
| Grandfather Passage |
| Multisyllabic Word Repetition |
| Sentence Repetition |
| Oral Mechanism Exam |
| Nonspeech Oral Motor Exam |
| Voice Evaluation |
| **Semantics** |
| Pyramids and Palm Trees (word) |
| **Phonology** |
| Phonological Manipulation Battery |
| **Repetition** |
| Word and Pseudoword Repetition |
| Bayles Sentence Repetition |
| **Syntax and Morphology** |
| Syntax Comprehension |
| Syntax Production |
| Inflectional Morphology |
| **Reading and Spelling** |
| Word Reading (high/low frequency, regular/exception) |
| Word Spelling (high/low frequency, regular/exception) |
| **Other** |
| Famous People Battery |

# Supplementary Materials: Determination of bilingual status

We used the following criteria to discern bilingualism status and establish proficiency in the language:

- They reported using the language as a fundamental part of their job (e.g., translator, language teacher, or other indication that they used a second language at work)
- They reported use of a different language in the home environment (speaks a certain language at home with family, lived/worked in a different country)
- There was indication that the neuropsychological evaluation was conducted in English,

but that English was the individual’s second language (therefore the individual is proficient in English as well as another language)

- They were educated partly in another country wherein the language of education was reported to be different than their second language and may have reported continuing to use the language of education with family/friends
- Individuals were excluded if they only took classes in the language and did not engage in speaking the language outside of that setting
